# Mobile App–Supported Self-Management for Chronic Low Back Pain: Realist Evaluation

**DOI:** 10.2196/66435

**Published:** 2026-02-17

**Authors:** Rebecca Hunter, Trish Gorely, Michelle Beattie

**Affiliations:** 1 Centre for Rural Health Sciences, University of the Highlands and Islands, Raigmore Hospital, Old Perth Road, Inverness, IV2 3JH, United Kingdom, 44 01463 279805; 2 School of Communities and Education, Northumbria University, Newcastle, United Kingdom

**Keywords:** chronic low back pain, back pain, realist evaluation, self-management, mHealth, mobile health, mobile app, smartphone, digital health, digital technology, digital intervention

## Abstract

**Background:**

As the world’s population ages, the prevalence of chronic low back pain (CLBP) is increasing, placing a substantial burden on individuals and health care systems. Mobile health (mHealth) apps offer a potentially scalable solution to support self-management, but little is known about how, why, for whom, and under what circumstances such tools work in real-world settings.

**Objectives:**

This study aimed to test and refine 3 program theories—developed through a previous realist review—on how mobile apps support CLBP self-management. The goal was to understand the key contextual factors and mechanisms that influence when and why a digital self-management intervention may succeed or fail.

**Methods:**

A realist evaluation was conducted using one-on-one telephone interviews with 9 participants who had used the Curable app for 3 months to self-manage their CLBP. Realist interviews followed a teacher-learner cycle to explore, test, and refine the program theories. Abductive and retroductive analysis was used to develop context-mechanism-outcome configurations (CMOCs), which were synthesized into refined theories of digital self-management in chronic pain.

**Results:**

A total of 20 CMOCs were constructed, supporting 3 overarching program theories centered on empowerment, self-management burden, and timing. First, the app was empowering when it offered credible and accessible knowledge that helped participants understand their pain, build confidence, and reduce reliance on health care providers. However, engagement depended on individual beliefs and expectations: those with strong biomedical views struggled to connect with the app’s psychosocial framing. Second, while the app could ease the burden of self-management by offering support between appointments, it could also increase burden during flare-ups, when users lacked the capacity to engage. Features such as proactive content delivery and low-demand interfaces were viewed as essential for continued use. Third, timing emerged as a key factor. Early introduction was beneficial for some, but others needed to first accept the chronicity of their condition before they were ready to engage with self-management tools. Trust in the source recommending the app also influenced engagement. While clinician endorsement was often valued—especially early in the self-management journey—participants who had experienced unmet needs or disillusionment in clinical encounters reported that peer recommendations or nonclinical sources held greater weight. This highlights the importance of aligning recommendations with individuals’ evolving relationships with authority and trust.

**Conclusions:**

Mobile apps like Curable (Curable Inc) can support empowerment and continuity of care in CLBP, but their success depends on personalization, timing, and relational dynamics. To prevent feelings of abandonment, such tools should be introduced as an adjunct to—rather than a replacement for—ongoing clinical support.

## Introduction

In 2020 nearly 10% of the world’s population (approximately 619 million people) suffered from low back pain [1]. Studies estimate that between 5%‐15% of people will go on to develop chronic low back pain (CLBP; pain lasting longer than 3 months) [2,3]. People with CLBP are frequent health care users, which places a significant strain on medical services [3,4]. This demand is expected to rise as the global population grows older and the prevalence of CLBP increases with age [5]. Mobile health (mHealth) apps are becoming powerful tools in assisting health care services meet the rising demand of caring for people with chronic health conditions [6-8].

mHealth apps are software programs designed to be downloaded and run on smartphones or tablets with the aim of maintaining, improving, or managing the user’s health [9]. mHealth apps have been recognized as a promising way of delivering timely, cost-effective, and individualized care [10]. It has been suggested that using mHealth apps to treat people with CLBP could reduce the number of general practitioner visits, provide quicker virtual consultations, promote self-management, and improve the psychological impact of pain [11]. Studies suggest that self-management apps for CLBP can be effective in reducing pain and disability [12-15].

However, recent reviews evaluating mHealth interventions for self-managing low back pain highlight mixed findings and persistent challenges. While some studies report improvements in pain and function, others point to inconsistent evidence, low methodological quality, and poor engagement [16,17]. Many apps reflect a narrow biomedical focus, lacking personalization and theoretical underpinning, and often fail to incorporate psychological and social dimensions of pain management. This limits their potential impact, particularly for people with complex, long-term conditions like CLBP [18]. These limitations underscore the need for theory-led research to better understand how, for whom, and in what contexts mHealth interventions work.

The Medical Research Council (MRC) encourages a theory-based approach to evaluation when reviewing complex interventions [19]. Theories can help identify behavioral mechanisms and contexts likely to bring about a desired outcome and, in so doing, potentially increase the effectiveness of both the intervention and its implementation [20]. This type of research is arguably of greater value to policy makers and program developers as the results go beyond reporting on effect sizes, which may be difficult to translate into real-world implementation [21,22]. Realist evaluations are a theory-led approach to evaluation designed to provide guidance on how to implement a program in a particular setting while avoiding potential obstacles along the way [23].

This study addressed the gap in theory-based research on self-management apps for CLBP. Aligned with the MRC’s guidance on evaluating complex interventions, a realist evaluation was used to explore a commercially available self-management app. We selected Curable (Curable Inc.), a generic pain management app ranked among the top 3 in a systematic review of 19 chronic pain apps [24], notable for its embedded pain education—a key component of effective self-management [25]. Unlike apps specifically targeting musculoskeletal or low back pain, Curable’s broader biopsychosocial approach aligns with current understanding that chronic pain, including CLBP, requires multifaceted management [26]. Rather than evaluating Curable’s effectiveness, the app was used as a case study to test and refine program theories developed in a previous realist review [27]. This study aimed to extend their applicability by evaluating these theories in a real-world setting with individuals living with CLBP. Ultimately, the evaluation sought to refine existing theories and contribute to a more robust framework explaining who benefits from a self-management app for CLBP, why, and under what circumstances.

## Methods

### Ethical Considerations

The study received ethical approval from the University of the Highlands and Islands Research Ethics Committee (ETH2122-0819) and was conducted in accordance with the principles of the Declaration of Helsinki. Written informed consent was obtained from all participants before data collection. Participants were informed of the nature and potential consequences of the study and of their right to withdraw at any time without consequence. All data were anonymized before analysis and confidentiality was maintained in accordance with UK data protection legislation (UK GDPR). While no honorarium was provided, participants were offered a 12-month subscription to the Curable app as a noncoercive form of compensation, allowing continued access beyond the 12-week study period should they choose to continue using the app.

### Theoretical Framework

Two substantive theories were used in this study to inform the testing and evaluation of the program theories: burden of treatment theory and empowerment theory. The burden of treatment theory, developed by May et al [28], focuses on the physical and emotional demands that patients face in managing chronic conditions, highlighting the work involved not only for the patients themselves but also for their relational networks (eg, family and health care providers). This theory was selected due to its emphasis on the complexity of managing CLBP within a health care system that increasingly expects patients to take responsibility for their care. It provided a useful lens for exploring how the structural, institutional, and professional dynamics interact with the experience of self-management in this context.

The empowerment theory used here draws from Lee and Koh’s [29] model, which combines both the behavioral and psychological dimensions of empowerment. This theory was selected because it helps to explore the role of health care professionals (HCPs) in empowering patients to take control of their self-management, aligning with the goal of understanding how digital health tools, like the Curable app, might influence patient empowerment in managing CLBP. These theories guided the development of program theories by providing conceptual frameworks to understand the interactions between health care systems, patient behaviors, and technology. They also helped to identify key themes in the data, particularly around the concepts of support, control, and autonomy, which were central to understanding how participants engaged with the app. The use of substantive theories in this way is typical in realist research, as it allows for a richer, more contextually grounded exploration of program theories and the mechanisms at play [30].

### Clarification of Key Terms

To ensure conceptual clarity and consistency throughout the review, we outline below how key terms have been defined and operationalized. This includes our understanding of context, mechanism, and outcome within a realist framework, as well as how we have defined self-management, which is central to the intervention under study.

The following key terms are used consistently in this paper and are defined as follows:

1. Self-management: our definition of self-management was informed by a number of key papers in the field of self-management of chronic conditions, including Barlow et al [31] Bodenheimer et al [32], Lorig et al [25], and Wagner et al [33]. In this study, self-management was defined as the ability of an individual—supported by health care providers, family, and community—to acquire the skills and confidence to manage their chronic condition on a daily basis. This process was considered dynamic, allowing individuals to adapt to the fluctuating nature of CLBP and the changing intrinsic and extrinsic factors in their lives.
2. Context: the dynamic and influential conditions or circumstances that shape how an intervention operates. Contexts are not static backdrops but active forces that determine whether and how mechanisms are triggered. These may include individual factors (eg, psychological state and previous knowledge), social or environmental conditions, or institutional settings, all of which influence how participants engage with and respond to the intervention.
3. Mechanism: the underlying processes that explain how and why an intervention works (or does not). Mechanisms involve the interaction between the resources provided by the intervention (eg, app features) and participants' responses to these resources (eg, changes in confidence, engagement, or self-efficacy). Mechanisms are context-dependent and are activated based on the specific conditions in which they operate.
4. Outcome: the results or effects produced by the intervention, which occur because of the activation of mechanisms within a specific context. Outcomes may include changes in behavior, attitudes, or health status.

The definitions of context, mechanism, and outcome used in this study are drawn from our previously published work on realist methodology [34]

### Study Design

A realist evaluation of a commercially available chronic pain self-management app was undertaken to test and refine program theories, derived from a previous realist review [27]. A realist evaluation is a systematic approach to studying how people respond to social programs delivered in real-life complex environments and how this influences program outcomes [35]. Realist logic is grounded in the concept of generative causation, which suggests that observable outcomes are brought about by underlying, often unseen, causal mechanisms that operate differently depending on context [36]. These mechanisms help explain why some people may benefit from an intervention and others may not. Context-mechanism-outcome configurations (CMOCs) are central to realist research, providing a way to understand how and why an intervention works (or does not) in specific contexts [37]. Another way to understand CMOCs is as testable propositions that explore the interaction between context, mechanisms, and outcomes, offering an evidence-based framework for explaining why different results might emerge in varying settings or populations [38]. Using a specific case study to test and refine theoretical understandings is a recognized and valuable application within realist research, particularly when evaluating complex interventions like mobile health apps for CLBP [39]. The aim of a realist evaluation is to develop theories as to who might benefit from an intervention or program, why, and in what circumstances.

The study was carried out remotely in Scotland with 9 participants living with CLBP. Participants were given licenses to use a commercially available app (Curable) for 12 weeks to help them self-manage their condition. No restrictions or limitations were placed on how the participants used the app. At the end of 12 weeks, participants engaged in one-to-one, semistructured realist interviews. The study adhered to the RAMESES II (Realist And Meta-narrative Evidence Syntheses: Evolving Standards II) reporting standards for realist evaluation [40] (refer to Multimedia Appendix 1)

### Intervention

The Curable app, developed by the US-based company Curable Inc, is a commercially available digital health intervention designed to support individuals in the self-management of chronic pain. It delivers content via a smartphone or web browser through a combination of audio lessons, written articles, and interactive exercises. The app’s program is grounded in pain neuroscience education and incorporates elements from cognitive behavioral therapy, mindfulness-based stress reduction, and expressive writing. Users are guided through the content by a virtual chatbot, which provides personalized recommendations and prompts based on user input. Interactive features include reflective journaling, symptom tracking, quizzes, and meditative or breathing exercises. The app encourages daily engagement, and its structure allows users to progress at their own pace through thematic content modules that address the psychological, emotional, and neurophysiological aspects of chronic pain [41].

### Recruitment

Participants were recruited through 3 concurrent channels. In the first stream, individuals who had participated in the previous realist synthesis and consented to future contact were emailed directly by the lead researcher. In the second stream, an online advertisement was posted on a study-specific Twitter account (@RealBackstory), and interested individuals responded via email. In the third stream, several pain charities and third-sector organizations shared the study advertisement on their websites and/or in their digital newsletters. Recruitment was entirely conducted online, with participants self-identifying by emailing the lead researcher to express their interest. As such, most participants were self-selected. However, to ensure diversity in experience and explore rival theories, purposive sampling was used to include a small subset (n=3) of individuals identified by a third-sector organization as having had particularly positive experiences with in-person self-management programs for CLBP. This purposive sampling allowed us to examine potentially contrasting perspectives, enhancing the explanatory power and trustworthiness of the findings [42].

### Eligibility Criteria

The inclusion and exclusion criteria are shown in Textbox 1.

Textbox 1. **The inclusion and exclusion criteria.**

Inclusion criteria:

- Any sex
- Aged 18 years or older
- Self-managing chronic low back pain (defined as pain in the lower back lasting >3 months)
- Have seen a medical practitioner about their back pain prior to being involved in this study
- Not receiving active medical treatment for back pain (surgery, physiotherapy, scans, etc)
- May still be taking prescribed analgesia for their low back pain
- Have a smartphone capable of running the Curable app
- Able to participate in a telephone interview

Exclusion criteria:

- Lacking capacity to provide informed consent
- Chronic pain condition that does not include low back pain
- Unable to commit to a 12-week study

### Data Collection and Analysis

In line with realist methodology, the number of interviews was guided by the principle of providing rich and relevant data for theory testing rather than aiming for saturation [43]. Data collection ceased once participants had contributed sufficient conceptual depth to meaningfully explore and refine program theories. This approach is consistent with realist evaluation practice [44] and supported by the concept of information power [45], which recognizes that the more relevant and information-rich the data, the fewer participants are needed.

One-to-one, semistructured realist interviews were conducted via telephone by the lead researcher (RH), a chronic pain specialist physiotherapist with qualitative interviewing experience and training in realist methodology. The realist interviews used a teacher-learner cycle, which is a key approach in realist evaluation [44]. In the teacher-learner cycle, the interviewer initially adopts a “teacher” role by presenting a program theory for the interviewee to consider. The interviewee then engages with the theory, drawing on their lived experience to confirm, refute, refine, or elaborate on it. Through this interaction, the interviewer becomes the learner, and the interviewee becomes a co-constructor of knowledge, actively contributing to theory refinement [46]. This cyclical dynamic enhances theoretical sensitivity and contributes to the trustworthiness of the realist research process [46] Interviews were audio recorded with consent and transcribed by a third party. The interview guide was based on the theories from a previous realist review (refer to Multimedia Appendix 2).

Data from the transcripts were analyzed to test the 3 program theories developed from the preceding realist review. These program theories were underpinned by 16 CMOCs; CMOCs are used in realist evaluation to explain how and why particular outcomes occur in specific contexts [34]. Transcripts were coded via repeated rounds of direct, indirect, and holistic coding to identify key causal mechanisms and contextual factors. This coding framework was based on training received from the Center for Advancement in Realist Evaluation and Synthesis (CARES; Jagosh, unpublished data, 2020).

Direct coding was used sparingly and involved a “cut and paste” approach, where brief but pertinent passages from the transcripts were extracted and added to a mind mapping software (XMind; version 22.10). These excerpts typically served as key quotes to support or challenge the CMOCs from the realist review. Indirect coding involved annotating sections of the transcript in the margins, with these annotations extracted and added to the mind map under the relevant CMOC. Each annotation was linked to the original data with participant ID and line numbers for transparency. Holistic coding examined larger sections or entire transcripts to create broader inferences, which were similarly extracted and placed in the mind map.

Inferences were made from the data using retroductive and abductive logic [47,48]. Retroduction refers to reasoning backward from observed outcomes or phenomena to theorize about the underlying causal mechanisms, often combining both inductive and deductive reasoning [48]. Abduction has been described as a form of “reading between the lines” to imagine the underlying causal mechanisms that are typically not directly observable [49]. Jagosh [48] refers to abduction as the creative process of generating inferences to hypothesize what might be happening beneath the surface. It involves reasoning based on “educated guesswork” and “informed hunches,” where researchers draw on existing knowledge to propose plausible explanations for observed phenomena.

The analytical process involved testing the CMOCs with both confirming and disconfirming data extracted from the transcripts. Confirming data referred to instances where the findings aligned with the proposed theory, supporting the link between context, mechanism, and outcome. Disconfirming data presented cases that contradicted the expected outcomes, challenging the initial theory. These contrasting pieces of evidence were placed side by side in the mind map to refine or adjust the CMOCs. Additionally, counterfactuals were explored—looking for instances where the absence of a certain context or mechanism led to a different outcome than expected. In cases where these contradictions or counterfactuals highlighted different theoretical possibilities, the CMOCs were split or revised to incorporate rival theories. This ensured the complexity of the data was preserved and not flattened out to create uniform themes [50]. The rigor of the final theories was judged using the principles of explanatory coherence [42,51] and was strengthened through an iterative comparison of evidence across participants. This involved assessing how well the findings aligned with existing literature and considering alternative explanations. Additional rigor was supported by ongoing discussions within the research team and peer review. While formal stakeholder engagement was not undertaken at this stage, informal discussions with a participant—who later became a Patient and Public Involvement (PPI) lead in follow-on research—also helped sense-check emerging interpretations.

### Realist Review Overview

A realist review was conducted before this evaluation to develop program theories about how and for whom self-management apps might work for people with CLBP. The review, published elsewhere [27] drew on 57 sources, including peer-reviewed literature, UK government policy documents, national pain charity resources, and nontraditional materials such as blogs, social media, and book chapters to capture a broad range of perspectives. To further enhance the depth and relevance of the findings, 19 realist interviews were conducted with people with CLBP, clinicians, and digital health designers. These interviews were integrated directly into the analysis to ensure that the voices and lived experiences of people with CLBP were central to theory development. The review generated 16 CMOCs, which were grouped into 3 overarching program theories: empowerment, self-management burden, and timing. These are summarized in Table 1 and referenced throughout the results section, where they were further tested and refined.

**Table 1.** Context-mechanism-outcome refinement.

Realist synthesis CMOCs^a^	After testing	Realist evaluation CMOCs	Sources	Example quote
CMOC 1. Convenience, accessibility, and choice Traditional NHS-led^b^ self-management programs for CLBP^c^ provide participants with little choice in how, when, and where they are delivered (C). A self-management app for CLBP can be accessed at a time and location that is convenient to the user (M), which restores a person’s sense of control and autonomy (O).	Refined	*CMOC 1. Choice and flexibility* When a person with CLBP has distinct preferences and varying levels of motivation for self-management (C), the app being adaptable and flexible enough to accommodate the user’s wants (I) leads to the patient experiencing a sense of agency, choice, and control over their self-management program (M), ultimately resulting in sustained use of the app and adherence to their self-management program (O).	Participants: 2,5,7,9	**...***having time to go out for a walk and listen to it* [the app] *was quite useful. But some of the content wasn’t great when you’re actually on the move*. [Participant 7] ...*it just didn’t suit me and my lifestyle*. [Participant 5]
*CMOC 2. Knowledge and self-reliance* Many people with CLBP rely on HCPs for support because they do not know how to manage their symptoms (**C**). By providing the user with knowledge, advice, and strategies to self-manage CLBP, a mobile app enables the user to gain confidence and agency (**M**) to manage their condition on their own (**O**).	Split	*CMOC 2. Knowledge is empowering.* For people with CLBP who feel dependent on health care professionals due to a lack of confidence in managing their symptoms independently (C), being offered an app that provides accessible information and practical self-management strategies leads them to feel more empowered and confident in their ability to manage their condition (M). As a result, they become more self-reliant and reduce their dependence on healthcare professionals (O). *CMOC 3. Knowledge reduces fear* For people with CLBP who experience fear and anxiety related to the uncertain causes of their ongoing pain (C), an app that provides education about CLBP can increase their sense of understanding, control, and agency over their condition (M), ultimately leading to greater confidence and engagement in self-management (O).	Participants: 2,3,4,6,7 Participants: 1,4,6,7	**...***I’ve gone to them* [GP’s] *looking for solutions an come away with nothing so I’m disappointed. Whereas when I found out about it* [pain] *myself, I could understand it more*. [Participant 3] ...*managing that fear is absolutely critical to pain management itself and an app can help with that, without a doubt*. [Participant 4]
*CMOC 3. Personalisation* A person with CLBP needs to be able to recognize themselves in the advice and information the app provides (**C**) so that they can trust what they are being told (**M**) otherwise they unlikely to engage with the app because they do not consider it as being relevant to their situation (**O**).	No change	*CMOC 4. Personalisation* For a person with CLBP to engage with the app, they need to see the advice and information as personally relevant to their situation (C). This recognition fosters trust in the app’s recommendations (M), which in turn leads to increased engagement because the individual perceives the app as a useful tool for managing their condition (O).	Participants: 1,4,5,6,7,9	*I’m not going to turn it* [the app] *on because I’ve started to hear more stuff that doesn’t mean anything to me. It doesn’t resonate with me*. [Participant 7]
*CMOC 4. Hope* If a mobile the app fails to provide the user with options that have not been tried before (**C**) then the initial hope they may have felt at being offered something that might alleviate their pain (**M**) can turn to bitterness, disappointment and sometimes anger (**O**).	Refined	*CMOC 5. Biomedical mindset* For people with CLBP who have a biomedical mindset (C), a self-management app that presents an alternative approach to understanding and managing CLBP may lead to frustration, annoyance, and disappointment (M), as the solution offered does not align with their expectations for managing their pain (O).	Participants: 1,5,6,8,9	*Pain is pain - the pain is not in my head – I have a diagnosis..I gave up on it because I just thought ‘this is ridiculous.’ I just felt that it wasn’t teaching me anything I didn’t already know. It wasn’t helping me.* [Participant 6]
*CMOC 5. Adjunct to care* If a self-management app was used as an adjunct to care and not a replacement (**C**) then HCPs^d^ are likely to welcome the tool as it helps them to deliver ongoing support remotely (**M**) thereby enabling them to treat more patients in their limited clinic time (**O**).	Refined	*CMOC 7. Enhancing Patient Confidence Between Appointments* Between healthcare appointments, when reduced contact can leave people with CLBP feeling vulnerable (C), giving them a self-management app can lead to the patient feeling reassured that they have timely and convenient access to ongoing support and answers (M), ultimately increasing their confidence to go longer before they need to see a HCP (O).	Participants: 8,9	**...***it* [the app] *helps until they maybe get an appointment.until maybe they go and have a onceover with a professional or a doctor – absolutely, great!* [Participant 8]
*CMOC 6. Burden of care* If people with CLBP lack the internal and or external resources to engage with a self-management app (**C**) then this can cause further stress and frustration (**M**) which adds to their burden of having to manage long term back pain (**O**).	Split	*CMOC 8. Proactive support* For individuals with CLBP experiencing severe symptoms that inhibit their ability to engage mentally with tasks (C), an app that proactively selects and plays content they have found helpful in the past reduces cognitive fatigue and the stress of decision-making (M), thereby making it easier to engage with the app, particularly during flare-ups (O) *CMOC 9. Voice activation* For individuals with CLBP experiencing severe flare-ups that impair their ability to engage physically or mentally with tasks (C), an app with voice activation (resource) reduces physical effort and fatigue (M), enabling continued access to support even during extreme pain (O).	Participants: 2,7,9 Participants: 2,7,9	*...at times when you are in so much pain.you just want some sort of reassurance or someone to come with an idea, because you can’t think of anything for yourself*. [Participant 9] *Voice activation is probably the only chance you’ve got because sometimes the pain is so unbearable. the only way I could actually unlock it* [the app] *would be voice activation.* [Participant 7]
*CMOC 7. Monitoring* Monitoring their progress with a mobile app and sharing this data with a HCP (**C**) can help a person with CLBP convey more of a ‘complete picture’ of how they are managing their condition (**M**) and thereby improve the communication and quality of a healthcare consultation (**O**).	Refined New New	*CMOC 11. Improved communication* During healthcare consultations, when people with CLBP find it challenging to accurately recall and articulate the details of their condition and management (C), using an app to record and share this data with an HCP can lead to the patient feeling less stressed and more in control (M), which improves their ability to communicate and enhances the quality of the healthcare consultation (O). *CMOC 6. HCP buy-in needed* When a HCP does not see the value in the app’s recorded data or dismisses its utility (**C**) then this disempowers the person with CLBP by devaluing their contribution to the consultation (**M**) which can adversely affect the therapeutic relationship from the patient’s perspective (**O**). *CMOC 12. Monitoring is burdensome* For people with CLBP who are constantly aware of their pain and benefit from strategies that allow them to mentally switch off from it (C), repeated prompts by an app to record symptoms can disrupt this mental disengagement, adding an additional burden to self-managing CLBP and serving as a constant reminder of their ill health (M), which can worsen the pain experience (O).	Participants: 1,2,4,9 Participants: 1,4,7 Participants: 3,5	...*you have to make them* [healthcare professionals] *believe you, and it creates so much stress in that interaction, and if you were doing than on an app then you’re taking the stress out of it*. [Participant 4] ...*when I took that to my neurologist he just went ‘but how do I know that that is genuinely like the right information?*’” [Participant 1] I found it [monitoring] very difficult to do and it actually made the pain worse because I was focusing on it. [Participant 5]
*CMOC 8. Meaningful consultations* Using an app to record and share their data with an HCP (either before or during the appointment) (**C**) can maximize limited consultation time (**M**) and thereby reduce the frustration felt by patients’ valuable consultation is wasted bringing HCPs ‘up to speed’(**O**).	Refined	*CMOC 10. Making consultations more effective/efficient* In healthcare settings with short patient appointments (eg, 10 min) where patients face the burden of using that time to provide background information (C), using an app to record and share pertinent data with an HCP ahead of time can lead to the patient feeling a sense of reduced frustration due to the expectation of a more efficient and focused consultation (M), ultimately leading to a more satisfactory consultation (O).	Participants: 4,5,6,7,9	…when you go and see him [the GP], he’s got the information there [on the app]… so I think there’s an opportunity for the GP to use it and save them a lot of time and a lot of work. [Participant 9]
*CMOC 9. Abandonment* By providing support as well as a means by which to contact a HCP if needed (**C**) a self-management app provides users with the reassurance of a ‘safety net’ should they feel they need additional support (**M**) thereby mitigating feelings of abandonment (**O**). *CMOC 10. Safety net* A self-management app that enabled a person with CLBP to maintain contact with a HCP (**C**) can provide a reassuring ‘safety net’ (**M**) leaving them more confident to be discharged from the healthcare service (**O**).	Merged	*CMOC 13. Ongoing support* After discharge, a person with CLBP needs occasional reviews from a HCP in addition to a self-management app (**C**) to reassure them that they have not been left to manage their condition on their own (**M**) and prevent feelings of abandonment (**O**).	Participants: 1,3,4,5,	A follow-up with a real live person is always a good idea in my book, just to see how they’re getting on. I wouldn’t want them to be given the app and say ‘There you are, bye bye’. [Participant 3]
*CMOC 11. Social Isolation* In the absence of supportive ‘real life’ relationships (**C**) a self-management app with a chat bot feature that has been designed to communicate in a human-like way to offer comfort and reassurance (**M**) can help someone with CLBP feel less alone (**O**).	Split	*CMOC 14. Chatbot is supportive* For people with CLBP who experience social anxiety, fear of judgment, or find social interaction draining (C), the non-judgmental and socially undemanding nature of an app’s chatbot (Intervention Feature) can reduce feelings of shame, guilt, and loneliness (Mechanism), ultimately helping to reduce feelings of social isolation (Outcome). *CMOC 15. Chatbot is poor proxy* For people with CLBP who find connecting with fellow human beings to be supportive and emotionally beneficial (C), the lack of warmth and empathy from an app’s chatbot (Intervention Limitation) can lead to the patient experiencing the interaction as fake, cold, and devoid of meaning (Mechanism), making the chatbot an inadequate substitute for human support (O).	Participants: 1,3,4,7,8 Participants: 1,7,8	*...if you’ve been isolated for so long then the very act of meeting someone, getting to know them.can be quite intimidating.whereas I suppose something, you know, that is not quite real would be easier in some regards.* [Participant 3] ...*it’s the personal thing, that’s the difference.it’s* [the chatbot] *not real, you’re not seeing facial expressions of somebody, how they react to you. The A.I* [artificial intelligence] *it’s robotic basically.,it’s cold*. [Participant 8]
*CMOC 12. Acceptance* If a person with CLBP remains steadfast in their search to find a cure for their back pain (**C**) and are unwilling to accept an active role in self-managing their condition (**M**) then they are likely to be disappointed with a self-management app because it does not rid them of their pain (**O**).	Refined	*CMOC 16. Expectations of a cure* For people with CLBP who are still seeking a cure for their back pain (C), a self-management app may not meet their expectations (M), leading to disappointment and a lack of engagement (O	Participants: 2,3,9	*Once you accept it and adjust your lifestyle around it and work with it not against it, any sort of information you can gather through the app* [is helpful]*. But it’s acceptance. A lot of people won’t accept that they’ve got a long-term issue*. [Participant 9]
*CMOC 13. Believed* Before a person with CLBP can accept the need to self-manage their condition they need to feel that HCPs believe them (**C**). Feeling believed triggers a sense of reassurance that they have been taken seriously (**M**) which makes them trust the HCP (**O**).	Refined	*CMOC 17. A proper diagnosis* For a person with CLBP, receiving a diagnosis that clearly explains their ongoing pain (C) can trigger feelings of validation and understanding of their pain experience, both to themselves and others (M), which helps them accept their condition and increases the likelihood of engaging with a self-management app (O)	Participants: 1,5,6	*I think if someone had no real reason and no answers that would be a bit more different. Because, like, I’ve got the answers.I’ve accepted it* [their diagnosis] *so I think that’s the difference for me.* [Participant 1]
*CMOC 14. Trust* When a person with CLBP trusts a HCP (**C**) then they are likely to be more receptive (**M**) to HCP’s recommendation of a self-management app (**O**).	Refined	*CMOC 18. Trust the messenger* When a person with CLBP is recommended a self-management app by a trusted healthcare professional or someone they view as credible and supportive (C), this endorsement can reassure them of the app’s credibility (M), making them more willing to give it a try (O	Participants: 1,3,4,5	*When you have been written off effectively by the NHS and effectively abandoned by them, then you’re skeptical and maybe that’s held me back from benefiting from the app potentially*. [Participant 5]
*CMOC 15. Fear/Reassurance* When a person has been reassured that there is no serious spinal pathology, and they are not likely to do any harm to their backs (**C**) they become less fearful of movement (**M**) and are more likely to engage with the strategies offered by a self-management app (**O**).	Refined	*CMOC 19. Reassurance* For people with CLBP who have high levels of anxiety about potential serious underlying causes of their pain or who hold strong beliefs that imaging is necessary for diagnosis (C), an assessment from an HCP alongside radiographic imaging (Intervention/Resource) is sought to provide trust and reassurance that nothing sinister has been missed (Mechanism), before they feel comfortable and confident to use an app to self-manage their CLBP (Outcome).	Participants: 1,3,5,7,8	..*an app can’t actually see what’s going on inside your body. So if there is something else causing something, you many need to go for that scan or Xray.I think there’s a time and a place for it* [an app] *and probably later on down the path* [Participant 8]
*CMOC 16. Early in the Journey* Introducing a self-management app early on in a patient’s journey —whilst medical management and investigations are ongoing— (**C**) can provide reassurance and advice to help a person with CLBP return to everyday activities (**M**) and thereby reduce the risk of maladaptive behaviors developing (**O**).	Refined	*CMOC 20. Timing of introduction* When a self-management app is introduced after medical investigations are underway or completed — a point at which individuals with CLBP feel their condition is being taken seriously (C) — they are more open to engaging with self-management strategies (M), allowing them to begin managing their condition earlier and improve their quality of life sooner (O).	Participants: 1,2,3,4,6,9	*I think the way I would personally think to benefit from it* [the app] *most would be if someone said ‘okay, we’re starting the treatment, we’re going to do this and this.but your homework in the meantime is try and make a tiny bit of progress yourself with the help of this app.* [Participant 2]

a CMOC: context-mechanism-outcome configuration.

b NHS: National Health Service.

c CLBP: chronic low back pain.

d HCP: health care professional.

## Results

A total of 9 participants, aged between 35 and 70 years and all living with CLBP for a minimum of 3 years participated in the study. A total of 3 were female and 6 were male. The 16 CMOCs from the preceding realist synthesis were tested and refined by the realist evaluation to produce 20 CMOCs (see Table 1).

These 20 CMOCs were refined, but did not fundamentally alter, the 3 program theories identified in the preceding realist review [27]. The groupings of the CMOCs and subsequent program theories were informed by Burden of Treatment theory [28] and Lee and Koh’s conceptualization of empowerment [29]. The evaluation revealed deeper insights into contextual factors and causal mechanisms that explain who might benefit from a self-management app for CLBP, why, and in what circumstances.

### Program Theory 1: Empowerment

Program theory 1 (see Textbox 2), informed by Lee and Koh’s [29] model of empowerment, was developed from 7 CMOCs: choice and flexibility (CMOC1), knowledge is empowering (CMOC2), knowledge reduces fear (CMOC3), personalization (CMOC4), biomedical mindset (CMOC5), HCP buy-in is needed (CMOC6), and reduce HCP workload (CMOC7).

Textbox 2. **Program theory 1: empowerment**

People with chronic low back pain can feel empowered by a self-management app if the app is personal and relevant to their situation, can be accessed when and where they need it, and is presented as an adjunct to ongoing care.

Participants highlighted the importance of having choice and flexibility in how they accessed self-management support. While the app was designed to be convenient (CMOC1), many felt its rigid content delivery did not meet their situational needs. This refined the original concept of “convenience” by suggesting that flexibility in interaction style—not just availability—is key to fostering a sense of control and agency.

The app’s educational content was widely seen as a means of empowerment, particularly when it provided new insights or helped fill perceived gaps in HCP knowledge (CMOC2). Beyond information acquisition, knowledge also had an important emotional impact in that it reduced the fear associated with chronic pain and helped participants shift from passive coping to active self-management (CMOC3).

However, this process was moderated by the app’s capacity to personalize its content. Participants expressed frustration at the generic feel of the Curable app, noting that it did not sufficiently tailor responses to individual experiences (CMOC4). This was particularly problematic for users who expected content to reflect their specific pain history or emotional context.

Engagement with the app was also shaped by participants’ underlying pain beliefs. Those with a strong biomedical orientation struggled with the app’s psychological framing, interpreting it as invalidating their pain (CMOC5). By contrast, participants who had already begun to reconceptualize their pain found the app aligned with their thinking and were more likely to benefit.

A recurring theme across CMOCs was the continued importance of HCPs. Participants expressed a need for HCP buy-in and endorsement to validate the app’s credibility and reinforce its content (CMOC6). In addition, the app was perceived as potentially useful in reducing demand on HCPs by enabling patients to self-manage more confidently between appointments (CMOC7). However, this potential was conditional on the app’s responsiveness and the willingness of HCPs to engage with its outputs.

Together, these CMOCs refined Program Theory One by demonstrating that empowerment in self-management is not solely a function of delivering knowledge. It depends on how that knowledge is delivered, who it is validated by, and how well the system supports both user autonomy and relational trust.

### Program Theory 2: Self-Management Burden

Program theory 2 (see Textbox 3 ), developed from 9 CMOCs and informed by May colleagues’ [28] burden of treatment theory, explores how self-management apps can either alleviate or intensify the burden of managing chronic low back pain (CLBP). The theory was refined to highlight that this burden is shaped not only by external resources, such as digital access, but more critically by internal capacities, especially during periods of heightened pain.

Textbox 3. **Program theory 2: self-management burden**

If people with chronic low back pain (CLBP) have the capacity to engage with a mobile app, then it can reduce the burden of having to self-manage CLBP by providing ongoing support, facilitating communication with health care professionals, and mitigating feelings of abandonment.

During acute flares, participants described a significant drop in cognitive and emotional bandwidth, making any self-management activity—even app use—challenging. The evaluation introduced two new CMOCs to address this context: voice activation (CMOC8) and proactive support (CMOC9). Participants suggested that voice control could reduce interaction effort, while a more proactive app—one that nudges rather than waits for user input—could better meet their needs during these episodes of intense pain.

Beyond these flare-specific adaptations, several CMOCs addressed how the app’s design intersected with routine management and interaction with HCPs. A recurring theme was the desire for data collection and sharing functionality, which participants believed would enhance the efficiency and focus of medical consultations. This supported and refined the review’s theory that digital tools can reduce the frustration of updating HCPs during limited appointment time (consultations, CMOC10). Participants felt that pre-shared data would allow consultations to center on interpretation and planning rather than recounting.

This aligns with CMOC11, which focused on improving communication. The app was perceived as a potential aid in memory and articulation, allowing users to convey details that might be difficult to express during face-to-face consultations. Importantly, some participants valued objective data as a way to counteract perceived skepticism from HCPs when symptoms were not outwardly visible—reducing the pressure to “look sick.”

However, not all interactions with data were beneficial. For some, symptom monitoring (CMOC12) backfired. Participants who had used other apps with frequent tracking prompts described heightened focus on pain, emotional distress, and even symptom worsening. For these users, digital tracking increased—rather than reduced—the burden of self-management.

Participants also discussed the app’s role as a source of ongoing support (CMOC13), particularly following National Health Service (NHS) discharge. While Curable provided a sense of continuity, it was not viewed as a replacement for professional input. Many still desired periodic reassurance and oversight from HCPs, highlighting that an app alone was insufficient to maintain confidence or alleviate feelings of abandonment.

The app’s conversational agent, Clara, further divided opinion. For some, the chatbot was supportive (CMOC14)—a nonjudgmental, low-effort interaction partner that offered comfort without the social labor of human connection. In contrast, others saw Clara as a poor proxy (CMOC15) for genuine empathy. The absence of emotional nuance, spontaneity, and body language left these participants feeling disconnected, reinforcing the view that digital tools can offer support but rarely replace relational care.

Collectively, these CMOCs refine Program theory two by emphasizing that the burden of self-management is not only shaped by how much a person must do, but also how sensitively digital tools respond to their physical, cognitive, and emotional capacity. Apps must strike a careful balance: offering timely, tailored, and low-effort support while avoiding features that may inadvertently intensify distress or isolation.

### Programme Theory 3: Timing

Program theory 3 (see Textbox 4), informed by both burden of treatment theory [28] and the empowerment model, emphasizes that the timing of introducing a self-management app is a key factor in determining whether it is embraced by users. The theory is underpinned by five key CMOCs: expectations of a cure (CMOC 16), a proper diagnosis (CMOC 17), trust the messenger (CMOC 18), reassurance (CMOC 19), and the early stage in the journey (CMOC 20).

Participants reflected on how the NHS, historically centered around finding cures and fixing ailments, shaped patients’ expectations. This cultural backdrop led to an assumption that health issues like CLBP should be resolved, rather than managed over the long term. For many patients, this expectation made it difficult to accept self-management solutions like mobile apps. However, participants noted that once individuals moved beyond the expectation of a quick fix, typically after receiving a thorough diagnosis and reassurance, they were more receptive to long-term management strategies, such as using a self-management app (CMOC 16).

Textbox 4. **Programme theory 3: timing**

A person with chronic low back pain is likely to benefit from a self-management app early on in their patient journey but not before they feel believed and reassured by health care professionals and have accepted their condition cannot be cured (CMOCs 16-20).

This shift in mindset was facilitated by key moments in the care journey. First, receiving a “proper” diagnosis—a concrete explanation of their pain—was seen as vital for helping patients accept their condition (CMOC 17). However, participants stressed that this diagnosis alone was not enough; they also needed reassurance from a HCP to ensure their pain was not indicative of something serious (CMOC 19). Only when these needs for validation and reassurance were met did participants feel ready to consider self-management tools, such as the app.

The introduction of the app was considered most effective when it occurred after these key steps had been completed. Timing was seen as especially critical—if the app was introduced too early, before a proper diagnosis or reassurance, it could be perceived as a “brush-off” or a superficial solution. But when the app was introduced at the right moment—typically after a thorough clinical assessment and reassurance—it was viewed as a supportive tool that could help users build self-management skills earlier, improving their quality of life sooner (CMOC 20).

Trust also played a significant role in whether participants engaged with the app. As highlighted in CMOC 18, recommendations from a trusted individual—often, though not always, a HCP—were more likely to lead to acceptance. However, when trust in HCPs was eroded due to unmet needs for validation or reassurance, some participants sought advice elsewhere, including from peers or alternative sources, who then became the preferred recommenders. This underlines the importance of introducing the app at the right moment, and through the right source, based on the patient’s journey and their current relationship with healthcare professionals.

Together, these CMOCs refine Program theory three by emphasizing that successful engagement with self-management apps is contingent on timing—not simply early in the journey, but at the right point in a person’s clinical and emotional trajectory. This includes when expectations of a cure have softened, when the individual feels their pain has been properly explained and validated, and when reassurance has been offered by a trusted source. The refined theory shifts away from a generic call for early intervention and instead highlights that the effectiveness of app-based self-management depends on readiness, which is shaped by how the health system meets core psychological and relational needs.

## Discussion

### Principal Findings

The aim of this realist evaluation was to test and refine 3 program theories developed during a preceding realist review on the use of mobile apps for the self-management of CLBP. These program theories centered around the concepts of empowerment, self-management burden, and timing, and were each underpinned by a set of CMOCs. They were informed by two key substantive theories: May and colleagues’ [28] burden of treatment theory and Lee and Koh’s [29] model of empowerment. Using the Curable app as a case study, this evaluation explored how, for whom, and under what circumstances such a digital self-management tool might work—or not—in everyday life. This study found that mobile apps like Curable can empower individuals with CLBP by providing accessible knowledge and reducing reliance on HCPs. However, the effectiveness of these tools is contingent on personalization, timing, and relational dynamics with health care providers. The success of such tools also depends on their introduction as an adjunct to ongoing care rather than a replacement, with careful consideration given to users’ evolving trust and readiness for self-management.

Our evaluation showed that digital self-management tools like the Curable app can support empowerment in people with CLBP by providing accessible and credible knowledge that fosters confidence, agency, and a reduced reliance on HCPs—a finding that echoes Lee and Koh’s [29] model of empowerment and aligns with Lim and colleagues’ [52] work showing that people with CLBP want information to manage their condition. Knowledge provided via the app helped participants understand their pain and reduce fear, confirming the empowering potential of education in chronic pain management [25]. However, empowerment was not universally experienced. Participants with a strong biomedical mindset struggled to engage with the app’s psychosocial framing, perceiving it as invalidating or insufficient—a reflection of the enduring influence of the biomedical model in shaping patient expectations [53]. This supports findings by Stenner et al [54] and Van de Velde et al [55], who argue that acceptance of pain is a prerequisite for engaging with self-management. These results highlight the need for apps to be personalized, both in content and delivery, to match users’ readiness for behavioral change—a key mechanism of engagement reflected in the technology acceptance model [56,57] and the unified theory of acceptance and use of technology [58].

Participants also believed that the app could help them rely less on HCPs between appointments, particularly when they felt the information was relevant and trustworthy—suggesting a role for digital tools in extending continuity of care. However, when HCPs failed to acknowledge app-related efforts or patient-generated data, participants felt dismissed, leading to disempowerment. This reinforces Lee and Koh’s [29] assertion that empowerment is relational and shaped by the behavior of those in power. For digital tools to fully realize their empowering potential, HCPs must validate and incorporate these tools into consultations. This aligns with existing research, which demonstrates that apps designed to track and share health data have improved consultations in the management of conditions like irritable bowel syndrome [59], chronic pain [60], and heart failure [61]. Taken together, these findings challenge the assumption that digital tools are inherently empowering and instead suggest their success is conditional on user beliefs, professional endorsement, and contextual fit. App developers should prioritize personalization and create tools that support a staged, user-centered journey toward empowerment, while health systems must formally recognize and accommodate digital self-management tools within routine care.

The evaluation highlighted that while self-management apps can ease the burden of care, they also risk amplifying it if poorly designed or insufficiently integrated with broader health care support. Findings align with May and colleagues’ [28] burden of treatment theory, which asserts that the capacity to engage with self-management depends on an individual’s personal resources and the demands placed upon them. Participants emphasized that during acute flare-ups of CLBP, the cognitive and physical effort required to interact with apps like Curable became a significant barrier. This underscores the importance of designing features that minimize interactional demands during times of distress—such as voice activation and proactive content delivery—echoing calls in the literature for “low-friction” digital health tools [62]. Although data-sharing capabilities were absent in Curable, participants voiced a strong desire for features that allow symptom tracking and integration into consultations, viewing the proactive sharing of health information with health care providers before consultations as beneficial. This aligns with Holt and colleagues’ [63] research, which demonstrated that previsit data collection enhances patient-provider communication, particularly in areas like respect, care, and perceived physician time, suggesting a pathway for better integrated health care support.

However, symptom tracking also revealed potential harms: for some, it became a persistent reminder of their condition, reinforcing pain salience [64]—a finding echoed in pain psychology literature cautioning against hypervigilance [64]. The app’s chatbot, Clara, was also polarizing. While some participants found its nonjudgmental, low-demand interaction helpful—aligning with its intended purpose—others felt it lacked the emotional depth of human connection, reflecting early observations in the literature on the limitations of artificial intelligence–driven relational agents in fully replicating therapeutic rapport [65]. However, more recent research suggests that humans can form meaningful emotional bonds with chatbots [66], which can help alleviate feelings of social isolation and loneliness [67]. This prompted participants to consider the potential for improvement: they envisioned that with more sophisticated programming and algorithms enabling greater empathy and responsiveness, a chatbot like Clara could potentially foster a more supportive relationship. Despite these possibilities, however, the majority of participants felt that the app alone was not sufficient for ongoing support after discharge from the health care service. While some found Clara helpful for building confidence between appointments, they agreed that a follow-up with an HCP was ultimately necessary. This perspective mirrors findings from a large cross-sectional online survey, which revealed that most people prefer using health apps as a complement to, rather than a substitute for, in-person doctor visits [68]. These results highlight that, although digital self-management tools can extend care between appointments, they are generally seen as supplementary to, not a replacement for, the ongoing expertise of health care professionals.

Program theory 3 focused on the role of timing in shaping a person’s readiness to engage with a self-management app for CLBP. While this theory was not explicitly framed around a single substantive model, it was informed by both the burden of treatment and empowerment frameworks. These concepts helped illuminate how the shifting emotional, cognitive, and relational context of living with CLBP influences when and how individuals feel capable of self-managing.

Our findings align with previous research, highlighting that patient engagement with self-management is more likely when individuals feel validated, reassured, and well-informed about their condition. For example, Toye et al [69] found that people with CLBP often struggle to engage with self-management until they have redefined their identity in relation to pain, a process that takes time and often requires credible explanation and validation from a trusted source. Similarly, Ong et al [70] found that people with sciatica were unable to emotionally adjust or cope with their symptoms until they received a credible diagnosis and explanation, which helped them make sense of their suffering. These findings echo the importance our participants placed on receiving a “proper” diagnosis and reassurance from an HCP before engaging with the app.

The concept of “timing” also reflects broader discussions in the literature around readiness for behavior change. Prochaska and DiClemente’s [71] transtheoretical model of change suggests that interventions are more effective when matched to a person’s stage of readiness—something that emerged clearly in our evaluation. Offering a self-management app too early, before patients feel they have been taken seriously or ruled out serious pathology, can create resistance rather than engagement.

Trust in the person recommending the app emerged as a key factor influencing engagement, aligning with Greenhalgh and colleagues’ [72] finding that patients are more likely to adopt health technologies when introduced by someone perceived as credible and caring. However, our study suggests that the timing of such a recommendation is equally important. For users early in their journey, a trusted clinician may be an ideal introducer. But for others who have experienced unmet needs, disillusionment, or frustration in clinical encounters, the same recommendation may be disregarded or even resisted. In these cases, peer recommendations or alternative sources carried more weight. This highlights that the right source of recommendation must come at the right time, tailored to the user’s current relationship with health care authority and trust.

While Program theory 3 does not rest on a distinct theoretical framework, its development was shaped by the same constructs underpinning the burden of treatment and empowerment theories. Specifically, it adds a temporal dimension—showing that empowerment and the capacity to shoulder treatment burdens are not static traits but unfold over time. Understanding when individuals are ready to self-manage is, therefore, essential in aligning support tools, like self-management apps, with patients’ evolving needs and expectations.

### Limitations

This study was guided by the RAMESES quality and reporting standards for realist evaluation [40] to ensure the research was undertaken with rigor and transparency. However, there are some limitations to be noted.

The Curable app did not have some of the functionality that was initially theorized to be beneficial, which meant some theories from the realist review could not be tested. However, to address this gap, participants used their experience with other health apps to help refine and develop theories.

Participation in the study was voluntary, which introduces a risk of selection bias. To address this, purposive sampling was used to ensure a variety of views toward self-management were represented. In addition, the study had a small number of participants and only evaluated the Curable app, which limits the claims that can be made about how representative its findings are for other self-management apps. However, the results have been presented as middle-range theories [30]. That is, the theories are at a level of abstraction whereby readers can judge whether they might be transferable to their context.

### Conclusions

mHealth apps have the potential to help people with CLBP self-manage their condition. This is important considering the growing number of people affected by this condition and the likelihood that the numbers will increase as the world’s population ages. This realist evaluation identified several key contextual factors and causal mechanisms to determine who may benefit from a self-management app and why. First, patient acceptance of their condition is important. Second, HCPs buy-in and personalized, adaptable content are essential for promoting sustained user engagement. Finally, to fully address patient needs, self-management apps should ideally be used in conjunction with ongoing support from HCPs. This integrated approach can help alleviate feelings of abandonment that may arise when solely relying on a mobile app.

## Supplementary material

10.2196/66435 Multimedia Appendix 1 RAMESES II reporting standards for realist evaluations.

10.2196/66435 Multimedia Appendix 2 Realist evaluation interview topic guide.

## Abbreviations

CARES: Center for Advancement in Realist Evaluation and Synthesis
CLBP: chronic low back pain
CMOC: context-mechanism-outcome configuration
HCP: health care professional
mHealth: mobile health
MRC: Medical Research Council
NHS: National Health Service
PPI: Patient and Public Involvement
RAMESES II: Realist And Meta-narrative Evidence Syntheses: Evolving Standards II
