# Supplementary material for: Mobile App–Supported Self-Management for Chronic Low Back Pain: Realist Evaluation
Source: JMIR Mhealth Uhealth. 2026 Feb 17;14:e66435. doi: 10.2196/66435 (PMC12912768; doi:10.2196/66435)
Supplement: Multimedia Appendix 2 [file mhealth-v14-e66435-s002.doc]

## Appendix 2: Realist Evaluation Interview topic guide

**Purpose**: To test and refine 16 CMOCs developed from a realist synthesis by exploring how they play out in participants’ real-life experiences of using the Curable app for CLBP.

**Structure**:

- Introduce each theory in plain, non-jargon terms.
- Encourage participants to reflect on their experience with Curable in relation to each CMOC.
- Use open-ended prompts to elicit C (context), M (mechanism), and O (outcome) elements.

**Warm-up Questions**:

1. Can you tell me a bit about your experience living with back pain?
2. Before using Curable, had you tried any other self-management strategies?
3. What were your first thoughts when you were introduced to the Curable app?

**Theory Testing Section (Organised by CMOC)**

Each theory is briefly explained to the participant, followed by discussion prompts.

**1. Control through Convenience**

“Some people find it easier to stick with an app like Curable because they can use it on their own time, in their own space.”

- Was that true for you?
- How did being able to use Curable when and where you wanted affect your experience?
- Were there times when convenience made a big difference—or didn’t matter?

**2. Knowledge Builds Confidence**

“Apps like Curable aim to give people useful knowledge and strategies so they feel more able to manage things on their own.”

- Did the information in Curable feel useful or empowering to you?
- Can you give an example of something you learned that helped?
- Were there things that confused you or didn’t seem relevant?

**3. Personal Relevance**

“Some people stop using health apps when the content doesn’t feel like it fits their situation or speaks to their experience.”

- Did you feel like Curable ‘got’ you and your experience?
- Were there things in the app that felt ‘off’ or not relevant?
- What made you trust (or not trust) what it was saying?

**4. Hope and Disappointment**

“For some, a new tool like Curable can spark hope—but if it doesn’t offer anything new, that hope can fade.”

- What were your hopes when you first started using Curable?
- Did it offer something different or new to you?
- Were there moments of disappointment?

**5. Supplement to Care**

“Some people find Curable most helpful when it works *with* support from healthcare providers—not instead of it.”

- How did Curable fit with any other care or support you were receiving?
- Did it feel like an extra layer of help, or something separate?
- Did any professional recommend it to you?

**6. Emotional Burden**

“Sometimes using an app can feel like more pressure—especially if you’re already dealing with a lot.”

- Did using Curable ever feel like extra work or stress?
- What made it easier or harder to keep going with it?

**7. Tracking Progress**

“Tracking your progress in an app like Curable might help you reflect—or help your doctor understand what’s been going on.”

- Did you use any of the tracking or journaling features?
- Did that information help you, or anyone else (like your GP)?
- Would you want to share that info with someone?

**8. Making Appointments More Useful**

“Some people use what they learn or track in the app to make healthcare appointments more productive.”

- Did Curable help you prepare for or reflect on medical appointments?
- Did it change what you talked about with your doctor?

**9 & 10. Feeling Supported / Safety Net**

“People sometimes feel more confident managing things on their own if they know they still have a safety net—a way to reach out if needed.”

- Did Curable give you a sense of being supported, or not being alone?
- Did you still feel like you could ask for help if needed?
- Were there times you felt on your own?

**11. Loneliness and Connection**

“Curable doesn’t have a social space, but it tries to offer comfort through the way it talks to you.”

- Did the tone or language in Curable help you feel less alone?
- Was there anything missing in terms of connection or community?

**12. Searching for a Cure**

“Some people are still hoping to find a fix for their pain—and that can make it harder to accept tools that focus on managing it.”

- When you started Curable, what were you hoping it would do?
- Did the idea of self-management feel frustrating or helpful?
- How did that change over time?

**13. Feeling Believed**

“Before people are ready to take on self-management, they often need to feel like professionals believe them and take their pain seriously.”

- Did you feel believed by professionals before using Curable?
- Did Curable reflect your experience in a way that made you feel seen or validated?

**14. Trusting the Recommendation**

“People often only try apps like Curable if someone they trust recommends them.”

- Who introduced you to Curable?
- Did their opinion make you more likely to use it?
- Did using the app change your trust in them?

**15. Fear and Reassurance**

“Some people are scared to move or do things in case they make their pain worse—but reassurance helps.”

- Did Curable help reduce fear about activity or movement?
- Was there a turning point when something helped you feel safer?

**16. Early Support**

“Getting access to tools like Curable early—before unhelpful habits or fears set in—can really help.”

- When in your journey did you start using Curable?
- Do you think it would have helped more or less if you’d had it earlier?
- How did your needs change over time?

**Closing Section**

- What was the most helpful or unhelpful part of using Curable for you?
- Looking back, what would you have changed about your experience?
- Are there any features or supports you wish had been included?
